# Umbilical Cord Matrix (Wharton Jelly) Mesenchymal Stem Cells in Next-generation Myocardial Repair and Regeneration: Mechanisms and Pre-clinical Evidence

**DOI:** 10.2174/011573403X372908250117092252

**Published:** 2025-02-25

**Authors:** Ewa Kwiecień, Marta Kot, Łukasz Czyż, Leszek Drabik, Adam Mazurek, Martyna Sikorska, Maciej Skubera, Łukasz Tekieli, Marcin Majka, Piotr Musiałek

**Affiliations:** 1 Department of Cardiac & Vascular Diseases, St. John Paul II Hospital, Jagiellonian University, Krakow, Poland;; 2 Department of Transplantation, Faculty of Medicine, Institute of Pediatrics, Jagiellonian University Medical College, Krakow, Poland;; 3 Department of Pharmacology, Jagiellonian University, Krakow, Poland;; 4 Department of Adult Congenital Heart Disease, St. John Paul II Hospital, Jagiellonian University, Krakow, Poland

**Keywords:** Wharton’s Jelly mesenchymal stem cells (WJMSCs), umbilical cord, stem cells, acute myocardial infarction (AMI), chronic ischemic heart failure (CIHF), myocardial repair, myocardial regeneration, animal models of human disease, advanced therapy medicinal product (ATMP)

## Abstract

Chronic ischemic heart failure (CIHF), caused by myocardial injury and cell loss, is a growing public health concern. Despite substantial investments in pharmaco- and device therapies for acute myocardial infarction and CIHF over the past decades, long-term prognosis has shown little improvement. There is a clear need to develop novel therapeutic strategies capable of attenuating progression from acute to chronic myocardial damage, reducing adverse myocardial remodeling, and enhancing myocardial contractility. Cell-based approaches are an important direction in basic and clinical research. Nevertheless, candidate cell types tested to-date in experimental and human studies show several fundamental limitations, including insufficient quantities and potency, poor myocardial uptake, immunogenicity and/or risk of tumorigenicity. Human umbilical cord matrix is a rich source of mesenchymal stem cells (Wharton’s jelly mesenchymal stem cells, WJMSCs). WJMSCs are naturally low-immunogenic, demonstrate high plasticity and proliferation capacity, and exhibit an absence of tumorigenic potential. Moreover, by producing specific anti-inflammatory cytokines and chemokines, they reduce the inflammatory response (hence their use in graft-*versus*-host disease) and have pro-angiogenic, anti-apoptotic, and anti-fibrotic properties, making them a natural player in myocardial repair and regeneration. Furthermore, WJMSCs can be expanded *ex vivo* with high genomic stability and full clonogenic potential and can be standardized as an “off-the-shelf” next-generation advanced therapy medicinal product (ATMP). This review aggregates essential, contemporary information on the properties and fundamental mechanisms of WJMSCs addressing the process of infarct healing and chronic myocardial injury. It discusses outcomes from pre-clinical studies, demonstrating improvements in myocardial function and reductions in fibrosis in animal models, paving the way for human ATMP trials.

## 1 INTRODUCTION

Cardiovascular disease will remain the leading cause of overall death and premature death in the coming decades, with acute myocardial infarction (AMI) and chronic ischemic heart failure (CIHF) as the main disease states [1, 2]. Data-driven prognoses show that CIHF will remain the number one cause of permanent disability, resulting together with its need for repeated hospitalizations in a major socio-economic burden [2]. Despite very substantial investments and research efforts over the last decades, today, the societal cost of cardiovascular disease remains substantially higher than that of cancer [2]. Although several major cardiovascular research endeavors of the last decades have produced new pharmacologic and device therapies (Table **1**) [3-41], those have not translated into major improvements in quality of life or survival in cardiovascular disease patients [1, 2, 42]. This is despite >50% reductions in age-standardized mortality rates for acute coronary syndromes occurring in high-income countries compared with <15% reductions in lower-middle-income countries of the world during the past 20 years [43]. One important reason is that the reduction in AMI deaths, seen with improved treatment strategies and networks, results in an increased number of individuals suffering from CIHF, posing a new challenge [44-46]. Overall, CIHF has a growing prevalence with very little change in prognosis over the years [42, 47]. In Europe, deaths from cardiovascular disease in those aged <70 years, commonly referred to as premature, remain a particular concern, with >60 million potential years of life lost to cardiovascular disease in Europe annually [48].

Therefore, the development of novel cardiac therapies with longer-term survival benefits is presently a major and largely unmet need [1, 2, 42, 49, 50]. The development (and implementation) of evidence-based new treatment approaches must be supported by consistent surveillance and monitoring so that the interventions can be appropriately targeted and evaluated translating into a public health benefit [48].

Major clinical trials that have determined contemporary clinical practice in acute myocardial infarction and heart failure with reduced left-ventricular ejection fraction and their key endpoints (Table **1**).

Cell-based reparative and regenerative approaches are today one of the fundamental research areas not only in cardiovascular medicine but also in other medical fields such as neuronal injury and neurodegenerative diseases (*e.g.*, Parkinson's disease, epilepsy, diabetes and gerontology) [1, 2, 51-53]. Regenerative medicine is anticipated to boost tissue repair and potentially provide effective replacement cell(s) and/or tissue(s) able to integrate into the ischemic zone(s) where, up till now, the damage has been considered “irreversible” [54].

First-generation myocardial regenerative approaches, although attractive conceptually for their simplicity, quickly faced a number of fundamental limitations, including not only insufficient quantities and potency of the therapeutic cell candidates but also their poor uptake in myocardial injury zone(s) [54, 55]. Meta-analyses of clinical studies employing 1st-generation stem cell therapies in AMI and CIHF demonstrate, at best, only mild benefits in the reduction of LV remodeling and/or increase in LVEF (with mesenchymal stem cells appearing more effective than bone-marrow mononuclear cells) and minimal/absent clinical benefit(s) in the context of heterogeneous, inconsistent and overall low-quality evidence [56-59]. This indicates the need to develop novel, more efficacious, cell-based strategies that should be subjected to rigorous (placebo/sham-controlled) appropriately-powered clinical studies employing blinded observer/observer-independent evaluation of cardiac function [60].

Novel, abundant sources of therapeutic cells, standardization of biological products, and improved delivery methods have been widely identified as key research targets in cell-based cardiovascular repair and regeneration [2, 13, 61, 62].

**Fig. (1):**
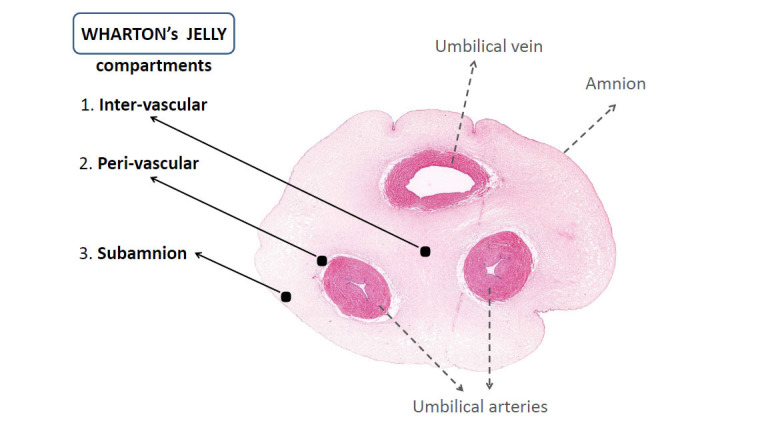
Umbilical cord cross section (according to Ref. [91], modified). It is worth noting that cells isolated from different compartments of the umbilical cord exhibit distinct properties. The vascular perivascular area contains highly differentiated cells, whereas the amniotic subchorionic region harbors immature cells with a high proliferative potential [94].

Mesenchymal stem cells residing in the umbilical cord matrix are a unique stem cell candidate that can address some important shortcomings of the typical 1^st^ generation cell sources: hematopoietic bone marrow cells showing poor regeneration capacity [55, 62, 63], and induced pluripotent stem cells were found tumorigenic [64-66] (Fig. **1**). The umbilical cord matrix mesenchymal stem cells, known as Wharton’s jelly mesenchymal stem cells (WJMSCs), possess unique features crucial for their potential therapeutic use [62, 67-75].

Importantly, WJMSCs do not express major histocompatibility complex (MHC) class II antigens and show a low expression of class I antigens. Thus, although allogeneic, WJMSCs are low-immunogenic, naturally overcoming a major disadvantage of other allogenic stem cell types considered for human use [76]. With their high plasticity, high proliferation capacity, and absence of tumorigenic potential, WJMSCs are today a particularly promising tool for the next-generation cardiovascular regenerative approaches [62, 70, 77, 78]. Evidence from WJMSCs characterization studies and from preclinical studies of WJMSCs use in the repair of acute and chronic myocardial injury [74, 79] position WJMSCs as a leading contemporary candidate tool in myocardial regeneration.

WJMSCs can be standardized as an “off-the-shelf” investigational (and potentially therapeutic) medicinal product (*i.e.* an advanced therapy medicinal product - ATMP^1^). The non-invasive harvest of WJMSCs (a “waste” material) and their reproducible expansion meet the highly desirable features of a cell-based biological product for use in ATMP clinical trials, hospital exemption administration, as well as for eventual routine clinical applications [2].

This overview provides a comprehensive knowledge base with regard to the biological and therapeutic properties of WJMSCs in their capacity to stimulate myocardial repair and regeneration. WJMSCs studies in small- and large-animal models of acute and chronic myocardial injury are discussed along with mechanistic insights into WJMSCs-mediated myocardial repair and regeneration, providing a foundational tool for clinical studies.

## 2 WJMSCS UNIQUE PROPERTIES

Stem cells are generally classified into embryonic stem cells, fetal stem cells, and adult stem cells [80]. Embryonic stem cells are pluripotent - they can give rise to tissues from the three germ layers [80]. Mesenchymal stem cells, depending on the type of source tissue from which they are isolated, can be classified as fetal or adult [80]. Mesenchymal stem cells are multipotent, and ability to differentiate into tissues from a particular germ layer. Mesenchymal stem cells are fibroblast-like, non-hematopoietic stem cells that efficiently proliferate *in vitro* (enabling their expansion) and may be re-transplanted *in vivo* [81]. Bone marrow [82-84] and several other fetal and adult tissues have been identified as an abundant source of mesenchymal stem cells [85-91]. One particularly attractive source of mesenchymal stem cells is the umbilical cord [67, 81].

The umbilical cord (Fig. **1**) is composed of two umbilical arteries and one vein that are surrounded by a mucoid tissue rich in proteoglycans and mucopolysaccharides and covered by amniotic epithelium [92, 93]. The mesenchymal stem cells of the umbilical cord matrix (WJMSCs) are trapped in the mucoid connective tissue during embryogenesis [67]. WJMSCs are present in the subamnion as well as in the intervascular and peri-vascular compartment of the umbilical cord (Fig. **1**). Subamniotic WJMSCs are the least differentiated and are considered to have greater proliferative potential [92-94]. Standardization of the WJMSCs isolation, characterization, and expansion is feasible, and it is critical on the path towards WJMSCs use as an ATMP therapeutic agent [95, 96].

As the umbilical cord is considered a waste tissue, WJMSCs harvesting - in contrast to embryo-derived stem cells - does not raise any ethical concerns. WJMSCs can be relatively easily isolated, harvested, and cultured [97]. With their fetal origin, WJMSCs represent a unique type of mesenchymal stem cell, characterized by their youthful properties such as higher proliferative potential, slower aging, and lower differentiation compared to adult mesenchymal stem cells. Since WJMSCs do not exhibit tumorigenic potential [98, 99], they can be safely used in regenerative medicine [100, 101].

WJMSCs completely fulfill all the criteria outlined for mesenchymal stem cells by the International Society of Cellular Therapy [102]. Specifically, WJMSCs (1) are plastic-adherent cells, (2) exhibit an elongated, spindle-shaped morphology (Table **2**), (3) express mesenchymal stem cells markers (≥ 95% expression of CD105, CD73 and CD90 as assessed by flow cytometry and ≤ 2% positivity for CD45, CD34, CD14 or CD11b, CD79a or CD19, and (4) demonstrate a three-lineage differentiation potential [67, 70, 92, 102-105].

WJMSCs can be reproducibly expanded to large quantities, making them available as an “off-the-shelf” cellular ATMP regenerative product [67, 106]. As fetal cells, they display high proliferative potential and delayed senescence. WJMSCs can undergo a slightly higher (50-70) number of divisions than the Hayflick limit for somatic cells (40 to 60) [107]. Moreover, the WJMSCs aging process (senescence) occurs much later compared to other mesenchymal stem cells, such as those derived from bone marrow or adipose tissue [108, 109]. WJMSCs, being ontologically young, retain their proliferative capacity for an extended period. The WJMSCs aging process and the loss of division capacity occur later, suggesting a greater 'efficiency' over a longer period. The number of divisions of WJMSCs may vary depending on culture conditions, donor age, and other factors [107-110].

WJMSCs exhibit the expression of several pluripotency markers including octamer-binding transcription factor 4 (OCT-4), SRY (sex determining region Y)-box 2 (SOX2), MYC proto-oncogene, bHLH transcription factor (c-MYC), homeobox protein NANOG, LIN28 protein, stage specific embryonic antigens (SSEA 1, 3, and 4), Kruppel-like transcription factor 4 (KLF4), teratocarcinoma-derived growth factor 1 (TDGF1), and zinc finger protein 42 (ZFP42); however, their expression levels are significantly lower compared to embryonic stem cells (ESCs) or induced pluripotent stem cells (iPS) [105]. These markers are consistent with the enhanced regenerative and differentiation potential of WJMSCs [67-69]. However, in contrast to pluripotent cells, WJMSCs are non-tumorigenic [70, 111]. Particularly important are the NANOG and OCT-4 factors that are crucial for maintaining the stemness state and the ability to self-renew [69, 70, 81, 92].

Importantly, WJMSCs exhibit elevated expression of early cardiac transcription factors such as kinase insert domain receptor (KDR), insulin gene enhancer protein 1 (Isl-1), and NK2 homeobox 5 transcription factor (Nkx2.5) [69]. The WJMSCs expression of these early cardiac transcription factors can exceed that of human embryonic stem cells [69]. WJMSCs express C-X-C chemokine receptors types 3 and 4, consistent with migratory and homing capabilities [69]. WJMSCs are markedly chemoattracted towards the ventricular myocardium, integrating robustly into the depth of ischemic cardiac tissue. These properties favor WJMSCs use in cardiovascular regenerative medicine [112].

The differences between WJMSCs and pluripotent stem cells are fundamental in the context of clinical use. The tumorigenic potential of embryonic or induced pluripotent cells has posed significant limitations in clinical applications [64, 113-116], thereby restricting their therapeutic potential [117].

One particularly important WJMSCs feature in the context of allogenic transplantation is their low immunogenicity [118-120]. Due to (1) low expression of MHC I molecules (that are normally present on the surface membrane of all nucleated cells in the human body and are responsible for the presentation of peptide fragments of proteins within the cell to cytotoxic T cells, triggering an immediate immune system response against any recognized “non-self” antigen); and (2) lack of MHC II molecule expression (normally found only on antigen-presenting cells and important in initiating immune responses), WJMSCs do not induce alloreactive lymphocyte proliferative response [118-120]. In addition, WJMSCs exhibit high expression of human leukocyte antigen G (HLA-G), a non-classical human leukocyte antigen class I molecule with strong immune-inhibitory properties [121]. HLA-G, typically present in trophoblast, is partially responsible for the tolerance of fetal tissue by the maternal immune system [121].

A number of studies have demonstrated the genetic stability of WJMSCs, defined as the absence of chromosome elimination, displacement, or chromosomal imbalance, and have shown that WJMSCs can be safely and reproducibly expanded *in vitro* [99, 122]. Moreover, WJMSCs are not susceptible to spontaneous malignant transformation *in vitro*, and no tumor formation has been observed in animal studies [97, 99]. Recently, Musiał-Wysocka *et al*. [70] investigated the safety of WJMSCs in comparison to induced pluripotent stem cells cultured in both normoxia and hypoxia and then injected into immunodeficient mice. The study confirmed that WJMSCs do not form teratomas *in vivo* even after culture in hypoxic conditions, whereas induced pluripotent stem cell-injected mice developed tumors, with histopathological analysis confirming typical teratoma morphology [65, 70].

Finally, WJMSCs share some properties with young fibroblasts (Table **2**) [123-128], making them a natural candidate for fibroblast replacement in biological processes involving these cells.

## 3 TISSUE REPAIR *VERSUS* REGENERATION

‘Repair’ is understood as mending tissue that is injured, damaged or defective. The process of repair suggests rebuilding a part of the loss without completely replacing it. Regeneration is a more advanced process of renewal and regrowth of the injured tissue or organ, involving the formation of a new tissue. The processes of myocardial repair and regeneration cannot be totally separated from each other, as they overlap and work together to improve heart function. Unlike repair, which often leads to scar tissue formation, regeneration restores the tissue to its original state with functional cells. In myocardial repair, while some aspects like inflammation and fibrosis are beneficial, excessive scarring can impair heart function. Regeneration offers the potential for restoring normal heart function after injury, as opposed to relying on scar tissue, which leads to impaired heart function over time. Promoting of the right balance between these two processes is important for restoring optimal heart function.

Fig. (**2**) [66, 67, 86, 105, 126, 129-140] provides a schematic presentation of mechanisms underlying WJMSCs reparative and regenerative capacities according to published evidence. Cell-to-cell interactions relevant to WJMSCs-mediated repair and regeneration are listed in Table **3** [85, 131, 141-151].

## 4 TYPES AND MECHANISMS OF MYOCARDIAL ISCHEMIC INJURY TO BE ADDRESSED BY CELL-BASED REPAIR AND REGENERATION

### 4.1 Acute Myocardial Infarction

Occlusion of an epicardial coronary artery causes acute hypoperfusion in the area supplied by the infarct-related artery, resulting in myocardial tissue damage. Without rapid reperfusion, most of the hypoperfused area becomes necrotic. The damage may extend to the total zone perfused by the occluded artery (area-at-risk). Prompt restitution of myocardial perfusion by pharmacologic and/or mechanical therapy salvages (at least part of) the area at risk from necrosis [152].

Human acute myocardial infarction (AMI) is typically associated with a loss of ≈10^9^ cardiomyocytes [153]. However, it is important to note that - given the cardiomyocyte to endothelial cell ratio in the mammalian heart (≈3:1) - AMI leads to a concomitant loss of ≈3x10^9^ other (mostly endothelial) cells [153]. The latter is relevant, as the repair/regeneration process needs to address the injury/loss of cells other than cardiomyocytes, namely the endothelial cells. The aim of cell-based regenerative therapies is to limit the loss and help rebuild the damaged tissue. However, the magnitude of the myocardial tissue loss due to myocardial infarction suggests that any substantial repair/regeneration of the loss is unlikely with the strategies employed so far.

Infarction results, in the first phase, from the acute reduction in oxygen delivery due to interrupted (or markedly diminished) blood supply. However, ischemic injury is inextricably related to another fundamental damage-causing mechanism that follows ischemia: reperfusion (hence “ischemia-reperfusion” injury). The reperfusion stage persists for several days. Reperfusion restores blood flow and oxygen provision to the ischemic tissue, a process that is in part beneficial and in part deleterious. The generation of reactive oxygen species enhances, through oxidative stress, endothelial and other cellular damage and stimulates inflammation [152, 154, 155]. It is important to recognize that while some myocardial cells die with ischemia, others die during reperfusion (lethal reperfusion injury) [156].

Necrosis triggers an extensive inflammatory reaction [157, 158]. Inflammatory cascades induce a cytokine storm, resulting in damage to cellular structures and cell death. The immune response to myocardial injury involves well definied players, such as neutrophils, monocytes/macrophages, dendritic cells, lymphocytes, and cardiac fibroblasts [154]. It is increasingly understood that immunomodulation during or after reperfusion may constitute an important therapeutic approach [154, 159]. Thus, the WJMSCs' immunomodulatory properties [92, 131, 160] may be particularly relevant in the context of reducing immune-modulated myocardial injury.

Although necrotic cell death is considered the leading mechanism of cellular loss in myocardial ischemia/reperfusion injury [152], several other forms of cardiac cell death have been recently reported to play a role in myocardial infarction. Those include apoptosis, autophagy, and necroptosis [154].

Necrosis is an uncoordinated and unregulated mechanism followed by an inflammatory response [152]. Typical morphological features of necrosis include contraction bands, karyolysis, mitochondrial swelling and disruption, membrane disruption in cardiomyocytes accompanied by microvascular destruction, interstitial hemorrhage, and inflammation [161, 162]. In contrast to necrosis, apoptosis, autophagy, and necroptosis are regulated pathophysiological processes that are controlled by specific signal transduction pathways [152].

Apoptosis is an energy-consuming form of cell death initiated by activation of sarcolemmal receptors such as FAS cell surface death receptor and tumor necrosis factor α (TNF-ɑ) as well as the mitochondrial release of cytochrome c, triggering a caspase cascade which results in intracellular proteolysis, without an inflammatory response [163].

Autophagy is another regulated process contributing to myocardial ischemia-reperfusion injury. Autophagy is characterized by lysosomal degradation and recycling of proteins [164]. The presence of double-membrane vesicles (autophagosomes) and increased expression of characteristic proteins are typical of an autophagic process.

Necroptosis incorporates hallmarks of necrosis and apoptosis [152]. However, necroptosis is a regulated process that needs activation by specific kinases, and can be inhibited by necrostatin [165, 166]. Although myocardial infarction has been considered primarily necrotic, the features characteristic of apoptosis, autophagy, and necroptosis are present in the infarct zone [152, 167].

Apoptosis is detected upon reperfusion in AMI cardiomyocytes and progresses up to 6 days post-reperfusion, in association with with infiltrated macrophages [168]. The Akt/mTOR/p70S6K pathway is also activated upon AMI reperfusion and remains elevated for up to 6 days (*p<*0.05). Ischemia activates the TLR-4-MyD88-dependent (cytokines/chemokines) and -independent (IRF-3) pathways in both ischemic and non-ischemic myocardium and remains high up to 6 days post-reperfusion (*p<*0.05) [168]. Accordingly, leukocytes and macrophages are progressively recruited to the ischemic myocardium (*p<*0.05). Ischemia up-regulates pro-fibrotic TGF-β that gradually rises collagen1-A1/-A3 mRNA with subsequent increase in total collagen fibrils and fibroblasts from 3 days post-reperfusion onwards (*p<*0.005) [168]. MMP-2 activity increases from ischemia to 3 days post-reperfusion (*p<*0.05). There is a timely coordinated cellular and molecular response to myocardial ischemia and reperfusion within the first 6 days after AMI [168]. Understanding of the mechanisms involved in tissue repair may facilitate the development of novel cardioprotective strategies.

Much effort has been invested in studying the molecular mechanisms underlying the development and progression of ischemia/reperfusion injury and post-ischemic cardiac remodeling [169]. Both during ischemia-reperfusion in the setting of AMI and during the chronic remodeling process following AMI, oxidative stress substantially contributes to cardiac damage [169]. Reactive oxygen species (ROS) generated within mitochondria are particular drivers of mechanisms contributing to ischemia/reperfusion injury, including induction of mitochondrial permeability transition or oxidative damage of intramitochondrial structures and molecules [169].

### 4.2 Infarct Healing

Tissue healing after myocardial infarction occurs through the activation of an endogenous repair response (endogenous myocardial reparation) [170]. Healing after myocardial infarction consists of three overlapping stages that include an inflammatory phase, a proliferative phase, and resolution [171]. Fundamental cellular players are neutrophils, monocytes/macrophages, dendritic cells, lymphocytes, and cardiac fibroblasts [154].

Coronary artery ligation results in pathological changes in cardiac muscle supplied by the infarct-related artery. Within 20 minutes, intracellular edema, swelling and distortion of the transverse tubular system, the sarcoplasmic reticulum and mitochondria are observed; these changes are reversible [155]. However, prolonged ischemia (~20 to 40 minutes) causes irreversible changes [172]. Irreversible damage occurs with its characteristic mitochondrial abnormalities such as swelling and internal disruption and margination of amorphous nuclear chromatin and relaxation of myofibrils [170].

In its early stage, myocardial injury triggers a local inflammatory response [158]. This leads to systemic inflammation, including stimulation of bone marrow-derived leukocytes and activation of complement, leading to the recruitment of neutrophils and monocytes at the infarcted area. Within 6-8 hours after AMI activated, neutrophils infiltrate into the ischemic myocardium, peaking at 1-3 days, and produce pro-inflammatory factors that attract monocytes [158].

**Fig. (2):**
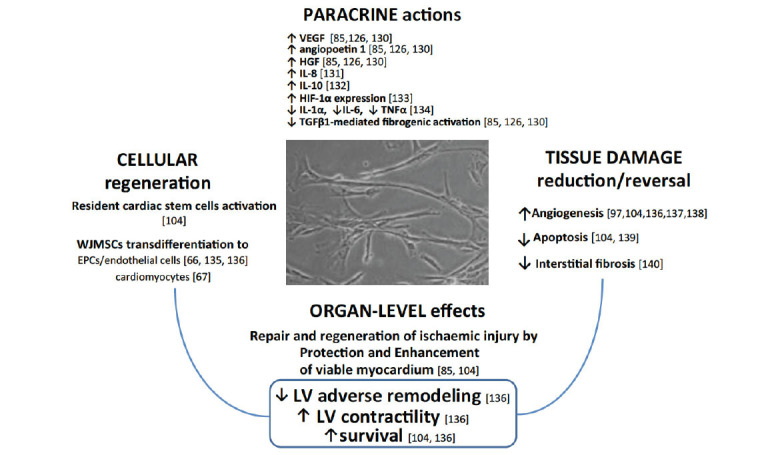
WJMSCs stimulation of myocardial repair and regeneration: fundamental mechanisms. **Abbreviations:** WJMSCs - Wharton’s jelly mesenchymal stem cells, EPCs - endothelial progenitor cells, VEGF - vascular endothelial growth factor, HGF - hepatocyte growth factor, IL-8 - Interleukin 8, IL-10 - Interleukin 10, HIF-1α - hypoxia-inducible factor-1α, IL-1α - Interleukin 1α, IL-6 - Interleukin 6, TNF-α - tumor necrosis factor α, TGF-β1 - transforming growth factor β1, LV - left ventricle.

Around the 3-7^th^ day of infarction, monocytes and macrophages infiltrate the border zone of the infarct [172]. At the injury site, monocytes differentiate into macrophages. There is disintegration of myofibers and phagocytosis and promotion of necrotic debris removal [172]. The monocyte infiltration is biphasic; pro-inflammatory monocytes predominate within the first 48 hours (peaking at days 3-4), whereas anti-inflammatory monocytes start to prevail 4-7 days later, peaking about day 7 [172]. M1 macrophages, predominant in the initial inflammatory phase, secrete high levels of pro-inflammatory cytokines, chemokines, and matrix metalloproteinases [170]. M2 macrophages, through their anti-inflammatory and pro-angiogenic properties, downregulate inflammatory response in myocardial infarction healing (Table **3**) [170, 173]. Infiltration of the infarcted area by B lymphocytes peaks at day 5 post-AMI and is responsible for pro-inflammatory response and mobilization of monocytes from bone marrow [174]. The ratio of pro-inflammatory *versus* anti-inflammatory macrophages is modulated by cytokines [175]. Activated monocytes and macrophages participate in crosstalk with other cells, including cardiomyocytes, fibroblasts, immune cells, and vascular endothelial cells. The pro-inflammatory response is crucial for wound repair, scar formation and compensatory hypertrophy after AMI [176], but a delay in alleviation of inflammation leads to myocyte hypertrophy, apoptosis and adverse LV remodeling, finally leading to heart failure [177]. The cytokine-mediated switch from the inflammatory to anti-inflammatory response - a process that can be mechanistically enhanced by WJMSCs at several levels (Fig. **2**) - plays an important role in reducing cardiac remodeling after AMI.

### 4.3 Role of Cardiac Fibroblasts in Myocardial Healing

Fibroblasts play a fundamental role in every phase of the healing response. They preserve the integrity of the extracellular matrix network, maintaining its geometry and function [178]. In the proliferative phase, fibroblasts transdifferentiate to myofibroblasts, which become the dominant cell type infiltrating the infarct border zone [179].

Moreover, fibroblast activation is coordinated with the inflammatory response via paracrine mechanisms [179]. They present a high proliferative capacity, express contractile proteins [157], produce higher levels of extracellular matrix proteins [170], and modulate matrix metabolism by expressing matrix metalloproteinases (MMPs) and their inhibitors [157]. These mechanisms protect the infarct zone against rupture and, by their contractile activity, retract the borders of the scar area, enabling wound healing [180, 181].

Fibroblasts interact with cardiomyocytes through several mechanisms [179]. These communications are essential for the myocardium to heal and recover [179]. Within the process of cardiac repair, the interaction between cardiac fibroblasts and cardiomyocytes is crucial for myocardium healing and recovery [179]. Downregulation of intestinal myofibroblasts occurs predominantly *via* inhibition of myocardin-related transcription factor A [147], providing an important mechanism for resolving the inflammatory processes (maturation phase). The maturation phase is characterized by the completion of collagen-based scar formation [147].

Apart from being a crucial determinant of cardiac repair, fibroblasts play an important role in long-term adverse remodeling [182]. The remodeling process can also be enhanced through the allogenic application of mesenchymal stem cells. WJMSCs share several properties with young fibroblasts [127], making them uniquely suited as a player in myocardial repair (Table **2**).

### 4.4 WJMSCs as ‘Natural’ Players in Infarct Healing and Repair

Evidence shows that WJMSCs are naturally chemoattracted towards the myocardial ischemic tissue and integrate with it [112]. A number of properties exhibited by WJMSCs are relevant to modulating the fundamental mechanisms on the interface of acute myocardial injury and healing, as well as in chronic myocardial ischemia injury (Fig. **2**).

**Fig. (3):**
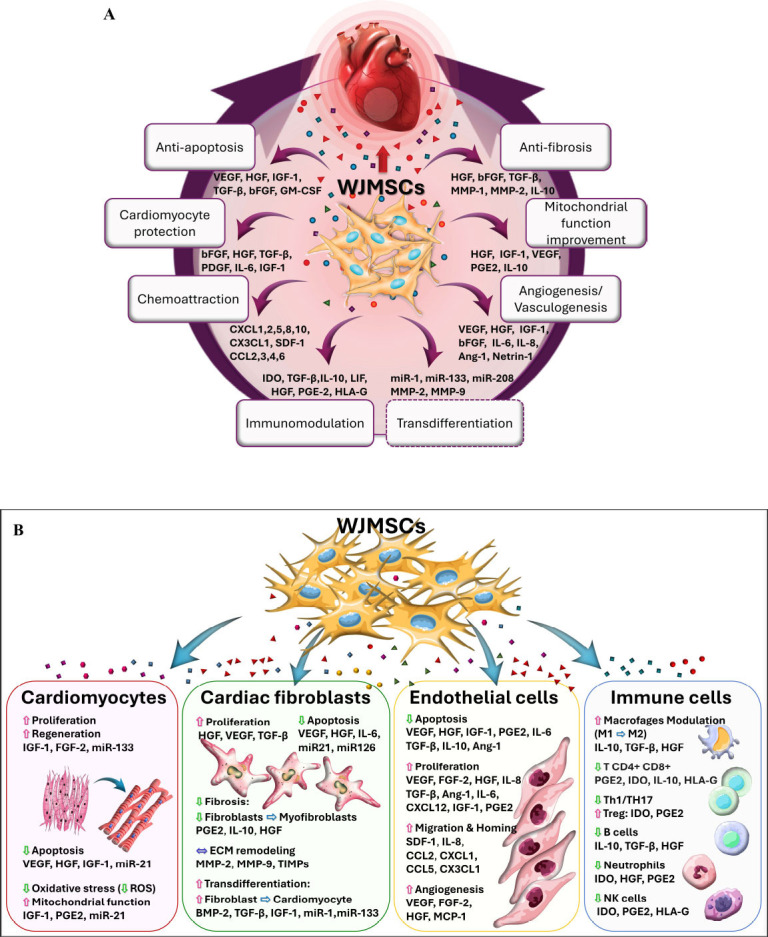
**(A)** Therapeutic properties of Wharton’s jelly mesenchymal stem cells driving cardiac regeneration. **(B)** Cellular and molecular mechanisms underlying the therapeutic effects of Wharton’s jelly mesenchymal stem cells in ischemic heart injury. (**A** and **B**) illustrate the paracrine mechanisms of action mediated by Wharton’s jelly mesenchymal stem cells (WJMSCs) in cardiac tissue regeneration following ischemic injury. The diagrams showcase the diverse therapeutic properties of WJMSCs, including the promotion of cardiac repair, inhibition of fibrosis, enhancement of vascular regeneration, and modulation of the immune environment to support tissue repair and functional recovery. WJMSCs exert their therapeutic effects on key cardiac cell types, including cardiomyocytes, cardiac fibroblasts, endothelial cells, and immune cells. WJMSCs promote cardiomyocyte proliferation and regeneration while protecting these cells from apoptosis and oxidative stress, leading to improved mitochondrial function. This protection is achieved through the downregulation of reactive oxygen species (ROS) and the enhancement of mitochondrial function, which supports the metabolic and energy demands of cardiomyocytes essential for recovery and improved cardiac performance. These mechanisms highlight the critical role of WJMSCs in preserving and repairing cardiomyocytes under ischemic conditions. WJMSCs support cardiac fibroblasts by enhancing their survival and proliferation, limiting fibrosis through the inhibition of fibroblast-to-myofibroblast differentiation, and facilitating extracellular matrix remodeling. Additionally, WJMSCs may induce the transdifferentiation of cardiac fibroblasts into cardiomyocytes, further contributing to tissue repair. Endothelial cells benefit from WJMSC activity through enhanced protection against apoptosis and the stimulation of angiogenesis and vasculogenesis. These processes involve promoting endothelial cell proliferation and migration, leading to the formation of new blood vessels that restore blood supply to damaged tissues. WJMSCs also play a pivotal role in immunomodulation by regulating immune cell activity. They shift macrophages from the pro-inflammatory M1 phenotype to the anti-inflammatory M2 phenotype, promote the activity of regulatory T cells, and reduce the pro-inflammatory activity of other T cell subsets. B cells and neutrophils exhibit decreased proliferation and activity, helping to mitigate inflammation, while NK cells reduce their cytotoxic responses. Finally, WJMSCs contribute to tissue regeneration by attracting progenitor cells and other reparative cell types to the site of injury through chemoattraction, facilitating their homing and integration into the damaged tissue. The molecules involved are indicated in the diagrams. **Abbreviations: VEGF** – Vascular Endothelial Growth Factor, **HGF** – Hepatocyte Growth Factor, **IGF-1** – Insulin-Like Growth Factor 1, **bFGF/FGF-2** – Basic Fibroblast Growth Factor 2, **TGF-β** – Transforming Growth Factor Beta, **IL-6** – Interleukin 6, **IL-8/CXCL8** – Interleukin 8 (Chemokine (C-X-C motif) Ligand 8), **Ang-1** – Angiopoietin-1, **PDGF** – Platelet-Derived Growth Factor, **MMP-1** – Matrix Metalloproteinase 1, **MMP-2** – Matrix Metalloproteinase 2, **MMP-9** – Matrix Metalloproteinase 9, **IL-10** – Interleukin 10, **SDF-1/CXCL12** – Stromal-Derived Factor 1, **CXCL1** – Chemokine (C-X-C motif) Ligand 1 (GRO-alpha), **CXCL2** – Chemokine (C-X-C motif) Ligand 2, **CXCL5** – Chemokine (C-X-C motif) Ligand 5, **CXCL10** – Chemokine (C-X-C motif) Ligand 10, **CX3CL1** – Chemokine (C-X3-C motif) Ligand 1 (Fractalkine), **CCL2/MCP-1** – Chemokine (C-C motif) Ligand 2 (Monocyte Chemoattractant Protein-1), **CCL3** – Chemokine (C-C motif) Ligand 3, **CCL4** – Chemokine (C-C motif) Ligand 4, **CCL6** – Chemokine (C-C motif) Ligand 6, **IDO** – Indoleamine 2,3-Dioxygenase, **LIF** – Leukemia Inhibitory Factor, **GM-CSF** – Granulocyte-Macrophage Colony-Stimulating Factor, **HLA-G** – Human Leukocyte Antigen G, **miR-21** – MicroRNA-21, **miR-133** – MicroRNA-133, **miR-1** – MicroRNA-1, **miR-126** – MicroRNA-126, **PGE2** – Prostaglandin E2, **BMP-2** – Bone Morphogenetic Protein 2, **TIMPs** – Tissue Inhibitors of Metalloproteinases, **M1** – Pro-inflammatory Macrophages (Classically Activated Macrophages), **M2** – Anti-inflammatory Macrophages (Alternatively Activated Macrophages), **Th1/Th17** – T Helper Cells Type 1 and Type 17, **Tregs** – Regulatory T Cells, **NK Cells** – Natural Killer Cells.

First, WJMSCs share several properties with young cardiac fibroblasts [128], making WJMSCs a natural player in the cardiac repair process [61, 74, 183]. WJMSCs transplantation into the infarct zone may exert benefit through at least 2 fundamental mechanisms, including (1) modulation of native fibroblast function and (2) fibroblast replacement. WJMSCs may affect native fibroblasts *via* the WJMSCs immunomodulatory effects (Table **3** and Figs. **3A**, **B**), resulting in downregulation of interstitial fibrosis [140]. Partial replacement of native fibroblasts with WJMSCs can also inhibit excessive inflammatory response and myofibroblast formation, improving internal scar formation balance (Fig. **2**). In addition, due to key genetic factors (that are absent in fibroblasts; Table **2**), WJMSCs are able to transdifferentiate into cardiomyocytes and endothelial cells [66, 67, 135, 136, 184, 185]. Nevertheless, transdifferentiation is rather unlikely to dominate the WJMSCS-mediated myocardial repair, indicating a leading role of WJMSCs immunomodulatory and paracrine mechanisms (Figs. **2** and **3A**, **B**).

WJMSCs - by their anti-inflammatory and immunomodulatory properties - may facilitate a switch from pro-inflammatory to anti-inflammatory mechanisms in the forming scar and thus exert a therapeutic effect by reducing scar formation, limiting the area at risk, and modulating the stunned myocytes so that they regain their physiologic contractile function [67, 104, 136, 157]. Evidence suggests that a switch from pro-inflammatory neutrophils (N1) and macrophages (M1) to their anti-inflammatory phenotypes (N2 and M2) may underlie an important part of the WJMSCs therapeutic effect [142, 186] (Table **3** and Figs. **3A**, **B**) [61, 71-76, 80, 89, 170, 184, 185, 187-206].

Overall, WJMSCs' immunomodulatory properties are consistent with enhancing the process of cardiac repair. In particular, WJMSCs secrete anti-inflammatory and immunosuppressive chemokines such as interleukin 10 and transforming growth factors β1 [75, 92, 131, 160] and suppress pro-inflammatory cytokines including interleukin 2 and interferon‐gamma [141]. Furthermore, WJMSCs - through their immunomodulatory properties and interactions with immune cells [142] - may alleviate the inflammatory response and inhibit myocardial fibrosis [139, 140]. Specifically, WJMSCs suppress T lymphocyte proliferation [46, 187, 188], inhibit the differentiation of T helper cells [142, 144], modulate T regulatory cell induction [142], and suppress NK cells [142] (Table **3**).

Along with their high proliferation capacity and release of large concentrations of chemokines, WJMSCs exhibit pro-angiogenic activity and modulate matrix remodeling [121, 189]. The WJMSCs-mediated reduction of inflammation [150], decrease in interstitial fibrosis [120, 150, 170], stimulation of angiogenesis [71, 72, 120, 150, 185, 190, 191] and preservation of cardiomyocytes indicate a unique role for WJMSCs in myocardial repair and regeneration (Figs. **3A**, **B**). Importantly, after WJMSCs are phagocytosed by monocytes, WJMSCS-derived extracellular vesicles may sustain therapeutic effect [192].

Finally, evidence from the murine model of myocardial infarction demonstrates WJMSCs promotion of wound healing by upregulation of genes involved in re-epithelialization TGF-β2 and neovascularization (HIF-1α) [193].

### 4.5 Chronic Ischemic Heart Failure

AMI (or repeated AMIs, including multiple small AMIs) underlie the development of CIHF. Scar formation and the loss of contractility cause major pathologic changes in function and structure of the left ventricle, termed “adverse remodeling” [194]. At the cellular level, myocardial remodeling involves alterations in myocyte biology, including stimulation of their adverse hypertrophy [195]. Pathologic changes in the extracellular matrix include initiation of interstitial fibrosis [195]. Cellular and molecular changes of cardiomyocytes and the surrounding interstitium result in the systolic and diastolic dysfunction of the left ventricular myocardium, underlying the symptoms and clinical presentation of congestive heart failure. Remodeling of the injured left ventricle after myocardial infarction due to volume and pressure overload results in increased wall stress, leading to macroscopic left ventricular dilation. Macroscopic changes include modification of left ventricular geometry: increased size, sphericity and left ventricular wall thinning [195].

Reduced ejection fraction and impaired contractility result from loss of contractile myocytes. The decrease in viable and correctly functional myocytes occurs through two major mechanisms, including necrosis and apoptosis [195]. Hypertrophic cardiomyocytes in the remodeled myocardium are susceptible to diffuse ischemia [194]. This, taken together with subendocardial blood flow reduction in the hypertrophic muscle, promotes subendocardial ischemic cell necrosis [196, 197]. Pressure and volume overload lead to neurohormonal stimulation, including the renin-angiotensin-aldosterone cascade, β-adrenergic stimulation, reactive oxygen species, inflammatory cytokines (*e.g.*, TNF-α), and mechanical stress. These activate the process of cardiomyocyte apoptosis [198-201]. Therefore, inhibition of pro-inflammatory processes is an important therapeutic target.

Molecular mechanisms underlying the development of CIHF, including metabolic alterations, reactive oxygen species overproduction, inflammation, autophagy deregulation, and mitochondrial dysfunction, have been recently reviewed in detail [169, 202]. Mitochondrial dysfunction is a key feature of CIHF [203]. Based on the mechanistic insight gained from rodent studies, the mechanisms for decreased mitochondrial oxidative capacity include altered mitochondrial ultrastructure, proteomic remodelling and oxidative damage of proteins and mitochondrial DNA, as well as impaired mitochondrial Ca^2+^ handling that accelerates the development of myocardial contractile dysfunction [203]. The transplantation of viable and redox-competent mitochondria has been proposed to improve myocardial recovery after ischemic damage [202], but a recent human translation of mitochondrial transplantation failed to provide consistent benefits [204].

## 5 WJMSCS-MEDIATED MECHANISMS OF MYOCARDIAL REPAIR AND REGENERATION

### 5.1 Paracrine Actions, Proangiogenic Capacity and Trophic Support

WJMSCs secrete paracrine factors, including cytokines, chemokines, and growth factors, which regulate stress-induced apoptotic pathways to enhance the survival of injured cardiac cells. WJMSCs release large quantities of pro-angiogenic factors, such as vascular endothelial growth factor (VEGF), stromal cell-derived factor-1 (SDF-1) and angiopoietin-1 (ANGPT-1), along with other angiogenic factors such as hepatocyte growth factor (HGF), TGF-β1, TGF-β2, basic fibroblast growth factor (bFGF), matrix metalloproteinases (MMPs), epidermal growth factor (EGF), platelet-derived growth factor-AA (PDGF-AA), and granulocyte colony-stimulating factor (G-CSF). These factors are crucial for initiating and maintaining angiogenesis [61, 71, 72, 97, 104, 122, 136, 137, 190, 205, 206]. WJMSCs also release pro-angiogenic chemokines from the CXC-chemokine family, including CXCL1, CXCL5, CXCL6, CXCL8 [85, 120, 144, 207, 208], and a number of other molecules documented to promote angiogenesis in animal models of myocardial injury (*e.g.,* VEGF, Netrin-1, Ang-1) [97, 104, 122, 136, 137, 144].

VEGF exhibits pleiotropic functions, promoting angiogenesis by supporting cell migration, proliferation, differentiation, and endothelial cell survival. Among the growth factors, VEGF appears to be the most critical for effective vasculogenesis (the formation of new blood vessels *de novo*) as well as angiogenesis (where new blood vessels form from pre-existing ones). VEGF secretion is stimulated by pro-inflammatory cytokines such as IFN-γ and IL-1β. VEGF overexpression by mesenchymal stem cells activates the SDF-1α/CXCR4 pathway and other mechanisms, including PI3K-NFκB, leading to the recruitment of pericytes and migration of cardiac stem cells into areas of infarction, stimulating angiogenesis. The elevated levels of VEGF exert anti-apoptotic and anti-hypertrophic effects on the ischemia-damaged cardiomyocytes [144, 185].

### 5.2 Transdifferentiation

The self-renewal capability of WJMSCs, their high proliferation rate, and their capacity for multilineage differentiation are well-documented [106, 209]. The multipotent character of WJMSCs enables their differentiation into derivatives of all three germ layers [66, 93, 98, 210-213]. Furthermore, there is evidence for WJMSCs capacity to transdifferentiate into endothelial cells [184] and cardiomyocytes [67]. When cultured with 5-azacytidine, WJMSCs transdifferentiate into cells expressing cardiomyocyte-specific proteins, including troponin I, troponin T, F-actin, N-cadherin, connexin 43, α-actin, GATA binding protein 4, and desmin [67, 69]. Recent studies in animal models have shown survival of the transplanted WJMSCs for several weeks after administration and their differentiation into cardiac-like cells expressing cTnT by immunohistochemistry [125] (Table **3**).

### 5.3 Immunomodulation

The immunogenicity of WJMSCs is known to be significantly lower compared to mesenchymal stem cells derived from other sources [118, 131, 142, 149, 159]. However, the mechanisms underlying the immunosuppressive properties of WJMSCs are complex and not yet fully elucidated. WJMSCs modulate immunity through both soluble factors and cell-cell contact [151]. WJMSCs do not express HLA-DR and co-stimulatory molecules such as CD40, CD80, and CD86 that are required for T-cell activation [103, 148, 188]. The absence of HLA class II antigens (including HLA-DR), along with the low expression levels of HLA class I antigens (HLA-A, HLA-B, HLA-C), allows WJMSCs to maintain their immune-privileged status, which helps them evade immune attack and minimizes the risk of rejection when transplanted into an allogeneic environment. This mechanism, combined with the immunomodulatory properties of WJMSCs, enhances their potential for allogeneic transplantation [103, 188, 214-217].

Through the secretion of indoleamine 2,3-dioxygenase (IDO), WJMSCs inhibit the differentiation of T follicular helper cells (Tfh), which leads to (1) the suppression of excessive immune responses; (2) the promotion of immune tolerance; and (3) the reduction of inflammation [85, 131]. The inhibition of Tfh differentiation by IDO helps prevent excessive antibody production, which can be important in avoiding autoimmune diseases or unwanted immune reactions, such as those following transplantation. IDO activity also contributes to the induction of immune tolerance, which is beneficial in transplantation (*e.g.*, to reduce graft rejection) and in treating autoimmune diseases, as it lowers the risk of immune system aggression against the body’s own tissues. IDO's action is associated with inhibiting the number of circulating pro-inflammatory Tfh cells, thereby reducing overall inflammation in the body and aiding in the treatment of various inflammatory conditions where an excessive immune response is particularly dangerous [186, 208].

Moreover, WJMSCs suppress the production of IFN-γ, stimulate the secretion of IL-10, and modulate the induction of T-regulatory cells [147-149]. The production of IL-6 by WJMSCs inhibits dendritic cells and induces them to adopt tolerogenic phenotypes [144, 214]. By producing prostaglandin E2, WJMSCs suppress the cytotoxicity of NK cells [144, 145] and inhibit the proliferation of CD4+ and CD8+ T-cells [144, 215].

WJMSCs have been successfully used in the treatment of graft-versus-host disease [216], particularly in steroid-resistant cases where standard glucocorticoid-based treatments fail to provide a therapeutic effect [217]. WJMSCs exert their immunosuppressive effects through multiple mechanisms: they inhibit the activation and proliferation of T-cells, suppress neutrophil adhesion to inflamed endothelium, and enhance the expansion of regulatory T-cells [218]. Recent data suggest that the immunomodulatory and anti-inflammatory properties of WJMSCs, administered iv. in doses ranging from 5 x 10^5^ to 3 x 10^6^ cells, may effectively counteract the cytokine storm arising from COVID-19 infection [219-222].

## 6 ROLE OF ANIMAL MODELS OF AMI AND CIHF IN ADVANCING CLINICAL RESEARCH

Pre-clinical evaluation of novel therapies for AMI and CIHF plays a pivotal role in advancing human trials. Animal models play a crucial role in generating the preclinical evidence enabling to perform Phase 1/2 human studies on myocardial repair and regeneration [104, 126, 136-139, 151].

While bench studies enable the investigation of cellular and molecular interactions, small-animal models (rodents, rabbit, guinea pigs) allow initial ‘proof of concept’ experiments [79]. Nevertheless, novel pharmacotherapies effective in rodents may fail to translate to humans [223]. Because of critical structural, functional, and molecular differences between small and large mammalian hearts, promising therapeutic approaches generally require preclinical testing in larger animal models before human translation [224] (cf., Table **4**). Several animal models of AMI and CIHF have been developed, with those based on ligation of the left anterior descending coronary artery (LAD) considered most relevant to human ischemic heart disease [225, 226]. Reproducibility of infarct size and LV remodeling in animal models usually allows to demonstrate the therapeutic effects of a new intervention with “n” numbers lower than those needed in human clinical studies.

Small-animal models serve as invaluable tools that have greatly advanced the treatment of myocardial disease, including the development of new treatments [203, 227, 228]. Despite their widespread use and acceptance, studies performed in small rodent models should nevertheless be interpreted with caution [203, 227]. Rodents, especially mice and rats, are powerful tools to study the mechanisms involved in the development of CIHF and novel therapeutic strategies. The human, mouse, and rat genomes have nearly the same size, each containing about 30,000 protein-coding genes, with about 99% of the genes encoded in the mouse genome having a homologue in humans [203, 227]. Further advantages of rodent models are the short breeding cycle and the availability of a variety of genetically engineered gain-of-function and loss-of-function models [203, 227]. Despite the specific limitations and differences outlined above, myocardial energetics and contraction are overall relatively similar between small rodents and humans. Consequently, numerous proteins share functions across species, which makes small rodent models inevitable tools to rapidly conduct proof-of-principle studies and to test different myocardial treatment strategies in AMI and CIHF [203, 227]. Rodents are typically on the same or very similar genetic background, which does not reflect the genetic heterogeneity of the patient population [203, 227]. Human ventricular myocytes predominately express b-myosin heavy chain, whereas adult murine cardiomyocytes mainly express α-MHC with rapid ATPase activity [203, 227]. Rabbit and pig show a greater than human potential for lethal arrhythmias in relation to acute myocardial ischemia [229]. The rat model of CIHF bears several shortcomings, including high mortality rates and limited recapitulation of the pathophysiology, etiology, and progression of human CIHF [230]. Furthermore, several differences comparing the small-animal and human hearts exist that result from the difference in heart rate (about 500-600 beats per minute in mice, 350 beats per minute in rats, 60-80 beats per minute in humans) [203, 227]. Advancements in magnetic resonance imaging and high-resolution transthoracic echocardiography enable today a detailed assessment of contractile function even in small rodents [203, 227]. Nevertheless, it is generally accepted that results from small-animal models require validation in large animals prior to trials in humans [203, 227].

Large animals (including pigs, sheep, and goats) are phylogenetically, physiologically and structurally closer to humans than rodents and therefore, at a molecular level, they have greater sequence homology with humans making interpretation of molecular events in large animals more relevant to man [79]. Aside from obvious similarities in size and physiology with humans, larger animals, such as pigs, are more clinically relevant models for studying the function/shape aspects of cardiac remodeling because the development of collateral blood vessels and the structural and functional alterations after AMI more closely recapitulate the human clinical phenotype [225, 226]. Large-animal models have provided significant advances in clinical practice [231]. Large animals are more similar physiologically and anatomically to man (size, tissue structure, and life span) and large animals are an ‘out bred’ population that more closely represents the heterogeneity of the human population than the ‘inbred’ small animal strains used in medical research [79].

Preclinical models of AMI and CIHF in large-animal models play a central role in providing new tools for early diagnosis and treatment [231]. Although economic costs, handling, personnel skills, and the necessary equipment are often limiting factors, large-animal models offer important advantages in terms of better clinical translation: they offer greater structural and functional similarity, and some models can also recapitulate the associated comorbidities [231]. LAD ligation is designed to affect large areas of the LV, so that later measurements of cardiac changes reach statistical significance without requiring large numbers of animals [225, 226]. In heart failure, large-animal models continue to be a mainstay for drug, cell, and gene therapy development as well as for surgical and minimally-invasive device development and procedure testing [224].

Swine is a prototypical large-animal model in pre-clinical evaluation of novel cardiac cell-based and device therapies [225, 231]. Porcine models of AMI and CIHF have the advantage of architecture and collateral circulation similar to humans, making it possible to predict and control infarct size and disease severity [225, 231]. Fundamental advantages of swine are a similar expression pattern of MHC isoforms and a similar reserve in heart rate and cardiac output compared to humans [203, 227].

Infarct size (IS; the AMI gold standard primary endpoint) and left ventricular geometry and function (wall thickness, WT; left ventricular dimensions and volumes; fractional shortening, FS; left ventricular ejection fraction, LVEF and remodeling indexes) are typical output measurements for *in vivo* studies of therapeutic interventions in small and large-animal models of AMI and CIHF [232-234] and -along with mortality/survival - they correspond to the typical endpoints in clinical trials in humans, as well as to cardiac parameters in everyday clinical practice (Table **1**).

Overall, studies in small-animal models importantly contribute to developing novel treatment strategies [203, 227] but large-animal models (in particular swine) have generally strong translational relevance to humans [223]. While small-animal models allow initial ‘proof of concept’ experiments, large-animal models allow clinically relevant assessments of safety, efficacy and dosing in cell-based therapies prior to clinical trials, and are thus indispensable in transition ‘from bench to bedside’ [79, 232]. Pre-clinical evidence demonstrates a consistent WJMSCs efficacy in small and large animal models of AMI and CIHF (Table **4**).

## 7 IMPROVEMENTS IN LV SIZE AND FUNCTION CORRELATE WITH CLINICAL OUTCOMES IN HUMAN PATIENTS: RELEVANT ENDPOINTS IN EVALUATION OF CELL-BASED STRATEGIES IN ANIMAL MODELS OF AMI AND CIHF

Recent analysis of data from 10 randomized clinical trials in AMI demonstrated that infarct size, evaluated by magnetic resonance or SPECT within 1 month after primary PCI, is strongly associated with all-cause mortality and hospitalization for heart failure within 1 year. Infarct size is, therefore, an important endpoint in clinical trials as a prognostic parameter [3], and infarct size reduction as is a key therapeutic aim in AMI.

In patients with LV dysfunction or heart failure after AMI, low LVEF is a ubiquitous risk marker associated with death [233]. A large-scale study of patients with stable heart failure followed up for 3-years demonstrated, among those with LVEF ≤45%, a near-linear reduction in mortality across successively higher LVEF groups (mortality of 51.7% with LVEF <15% *vs.* 25.6% with LVEF 36% to 45%, *p<*0.0001); an association relevant after multivariable adjustment [234]. Studies of ß-adrenolytic therapy in heart failure demonstrated that reduction in LV adverse remodelling is associated with improved long-term outcomes including survival [235] but the therapeutic effect magnitude may be dependent on myocardial viability [236]. Echocardiographic data from the Valsartan Heart Failure Trial showed that patients with worse LVEDD and EF are at highest risk for an adverse event yet appear to gain the most anti-remodeling effect and clinical benefit with treatment [237].

Recent meta-analysis of 30 mortality trials of 25 drug/device therapies (*n =* 69,766 patients; median follow-up 17 months) and 88 of LV remodeling trials of these therapies (*n =* 19,921 patients; median follow-up 6 months) in patients with LV dysfunction demonstrated that short-term trial-level therapeutic effects of a drug or device on LV remodeling are associated with longer-term effects on mortality [238]. In a contemporary registry of patients with heart failure with reduced LVEF, all-cause death or heart failure hospitalization occurred in 12% in the LVEF improvement group *versus* 25% in the group without an LVEF improvement (adjusted hazard ratio 0.50, 95% confidence interval 0.41-0.61) [239].

Overall, analyses of clinical data demonstrate that reduction in LV remodeling and improvement in LVEF are associated with reduction in mortality and adverse heart failure-related outcomes compared to patients with sustained LV systolic dysfunction [240].

This positions (1) infarct size reduction, (2) attenuation of LV remodeling and (3) LVEF improvement - along with increased survival (reduced mortality) - as relevant endpoints in pre-clinical and clinical studies of cell-based therapeutic strategies in ischemic heart disease.

## 8 THERAPEUTIC EFFICACY OF WJMSCS IN ANIMAL MODELS OF ACUTE AND CHRONIC MYOCARDIAL INJURY

A number of WJMSCs studies have been performed in small (rodents, rabbit) and large-animal models (swine) of AMI and CIHF [104, 126, 136-139, 151]. Details from those studies are provided in Table **4**, whereas summarized data are provided below. Overall, in both small- and large mammalian models, there is considerable pre-clinical evidence for enhanced myocardial repair and reduced left ventricular remodeling with human WJMSCs used to counteract acute and chronic myocardial ischemic damage.

### 8.1 Acute Myocardial Injury

Studies in animal models of AMI have consistently found improvement in left ventricular function with WJMSCs transplantation (Table **4**). First, Yannarelli *et al*. [138] evaluated left ventricular contractility in a mouse model of AMI comparing placebo administration (phosphate-buffered saline, PBS), bone marrow mesenchymal stem cells and WJMSCs, administered by intravenous or peri-infarct intramyocardial injections. Transthoracic echocardiography was performed by a blinded observer at baseline and 14 days after treatment. Left ventricular fractional shortening (LVFS) was significantly better in animals receiving peri-infarct cell transplantation compared with the placebo group (*p<*0.05). Importantly, the improvement was greater in mice administered with WJMSCs than in those receiving bone marrow mesenchymal stem cells. In contrast, the groups receiving cell treatment by intravenous injections showed no benefit from cell-therapy occurred, indicating that the intravenous delivery may be suboptimal [138].

Another study in a mouse AMI model compared peri-infarct injections of human WJMSCs with placebo (bovine serum albumin in PBS) injections [137]. Cardiac function was assessed by echocardiographic examination at baseline and 14 days after cell transplantation. WJMSCs-treated animals showed improved left ventricular ejection fraction (LVEF) and LVFS. There was also inhibition of remodeling, expressed by a reduction in left ventricular end-diastolic diameter (LVEDD) and end-systolic diameter (LVESD) (*p<*0.05 for all) [137].

Gaafar *et al*. [125] tested WJMSCs efficacy in a rat model of AMI. Four groups of animals were compared, including (1) control (non-manipulated) rats, (2) healthy rats injected with human WJMSCs, (3) AMI-induced rats, and (4) WJMSCs-treated rats on the third day after AMI induction. Cardiac function (LVEF) and LV dimensions were evaluated by echocardiography at baseline and 2 weeks after treatment. LVEF, LVEDV, and LVESV were significantly better in the WJMSCs-treated AMI group compared to the AMI group receiving no WJMSCs treatment (*p<*0.05). In addition, after 6 weeks, the survival rate in the AMI group that received WJMSCs was significantly improved in comparison to the control AMI group [125]. Overall, data from the study showed a benefit with human WJMSCs administration manifested by improvement in left-ventricular function and attenuation of left ventricle remodeling (Table **4**).

Latifpour *et al*. [241] evaluated WJMSCs efficacy in stimulating myocardial repair and regeneration in a rabbit model of permanent surgical left anterior descending (LAD) permanent ligation. Animals were divided into five groups: intact group, control group (the AMI model), PBS group (placebo administration in the AMI model), WJMSCs group (5 x 10^6^ cells), and 5-Azacytidine-conditioned WJMSCs group (5 x 10^6^ cells). Human WJMSCs were injected subepicardially 1 hour after AMI. Cardiac function was evaluated at 5 and 30 days after AMI. The authors showed a significantly greater improvement in left ventricular ejection fraction, fractional shortening and reduced scar tissue formation 30 days after AMI induction in animals treated with WJMSCs and 5-Azacytidine-preconditioned WJMSCs compared to those in the placebo-controlled group (*p<*0.05) [241].

The study by Lim *et al*. [151] tested allogenic (porcine) WJMSCs in porcine models of AMI. Mini-pigs, after surgical left anterior descending (LAD) artery ligation, were divided into three study groups: placebo (PBS), low dose (0.5 x 10^6^ cells/kg), and high dose (1.5 x 10^6^ cells/kg). Allogenic WJMSCs were delivered intravenously twice after AMI: 120 minutes and 4 weeks after LAD ligation. Cardiac function was assessed by echocardiography before, during, and after surgery and at 1, 4 and 8 weeks after infarct induction. These groups also underwent ^99m^Tc sestamibi myocardial perfusion single photon emission computed tomography (SPECT) and ^18F^-fluorodeoxyglucose (FDG) cardiac positron emission tomography (PET)/computed tomography (CT) at 1 week, 4 weeks, and 8 weeks after infarct induction. Transthoracic echocardiography demonstrated significantly improved LVFS at week 8 in the high-dose WJMSCs group compared to the PBS group (*p<*0.05) and a tendency for increased LVEF at 4 and 8 weeks in both low- and high-dose groups compared to PBS group. Moreover, M-mode images of 2D parasternal long-axis echocardiography showed improvement in left ventricular wall motion in both WJMSCs-treated groups at week 8 after AMI. SPECT and PET demonstrated a reduction of LV nonviable myocardium area in both the high- and low-dose WJMSCs groups compared to the placebo-treated animals (*p<*0.01). WJMSCs inhibited left ventricular adverse remodeling, as reflected by a marked reduction in fibrosis and reduced extracellular matrix deposition in the total myocardial area. Assessment of protein and gene expression levels showed a reduction of inflammation, reflected by decrease in inflammatory biomarkers (TNF-α and Interleukin-6). Connexin 43 expression in remote areas was greater with WJMSCs high dose in comparison to low-dose and PBS groups (*p<*0.05). Furthermore, the WJMSCs-treated animals demonstrated promotion of angiogenesis, as demonstrated by enhanced pro-angiogenic factors (VEGF and platelet/endothelial cell adhesion molecule 1) in the myocardial infarct and border area [151].

Zhang *et al*. [104] tested WJMSCs effects in a porcine model of AMI. Human WJMSCs, delivered *via* peri-infarct injections, were compared to placebo (PBS) after left anterior descending artery ligation. The study also involved a control group of AMI without cell or placebo injections. Cardiac function was assessed by echocardiography immediately after myocardial infarction and 6 weeks after therapy. WJMSCs administration to the infarct zone enhanced regional contractility of the infarcted area and improved global left ventricular function expressed as LVEF (*p<*0.001). Infarct-elicited deterioration in LV wall thickening was smaller in the WJMSCs-transplanted group compared with the PBS group (*p<*0.001) and smaller than in the group that received no cells and no placebo (*p<*0.001). Histologic evaluation of cardiac tissue specimens indicated WJMSCs-mediated enhancement of viable myocardium by inhibition of fibrosis and apoptosis in the infarct border zone (Table **4**) [104].

### 8.2 Chronic Ischemic Myocardial Injury

In a rat model of chronic ischemic myocardial injury, Wu *et al*. [136] compared WJMSCs with placebo (PBS) administration *via* peri-infarct injections. Cardiac function was assessed by echocardiography 2- and 4 weeks after treatment. At 2 weeks, the WJMSCs-treated group showed improvement in LVEF, whereas LVEF decreased in the placebo-treated control group (*p<*0.05). Left-ventricular diameters (both end-systolic and end-diastolic) were significantly smaller in WJMSCs-treated animals (*p<*0.05), consistent with effective inhibition of adverse remodeling. Moreover, myocardial thickening was also better in the cell-administered group (*p<*0.05). Importantly, the improvement was sustained 4 weeks after WJMSCs transplantation [136].

Liu *et al*. [139] tested the effect of human WJMSCs in a porcine model of chronic myocardial ischemia. Intracoronary cell administration of 30 x 10^6^ WJMSCs, followed by two additional intravenous infusions in the following 2 weeks, significantly improved LVEF compared to the placebo group (i.c. saline administration). WJMSCs-treated animals also showed better thickening of the infarcted wall, improved perfusion, and inhibition of left ventricular remodeling. Consistent with the functional data, histological evaluation showed reduced fibrosis and apoptosis in the WJMSCs-treated animals [139]. Note that the endpoints in animal studies of WJMSCs therapeutic efficacy in AMI and CIHF (Table **4**; LV contractility and size, attenuation of LV adverse remodeling, survival/mortality) are consistent with the endpoints in human studies driving contemporary clinical practice (Table **1**).

## 9 WJMSCS EFFECTS IN NON-CARDIAC TISSUES: MINIMIZING ISCHEMIC DAMAGE AND ENHANCING RECOVERY

Detailed characterization of the therapeutic potential of WJMSCs in enhancing the recovery of ischemic non-cardiac tissues is beyond the scope of this review. Nevertheless, it should be noted that studies in non-cardiac tissues are consistent with the WJMSCs stimulation of myocardial repair.

WJMSCs were demonstrated to alleviate the damage and promote tissue salvage in animal models of critical limb ischemia that poses an important medical and societal problem [242, 243]. In a mouse model of critical hind-limb ischemia (induced by femoral artery ligation). Shen *et al*. [132] compared three groups of animals (*n=*8 each): (1) ischemic mice, (2) diabetic ischemic mice treated with placebo (saline injections), and (3) diabetic ischemic mice treated with endothelial progenitor cells derived from human WJMSCs. WJMSCs cells (1 x 10^6^) were injected intramuscularly into the thigh muscle along the course of femoral artery within the surgery after artery ligation. Limb function assessment was performed using laser Doppler imaging at baseline and at 3 and 7 days after treatment. WJMSCs injections improved the ischemic to non-ischemic limb blood flow ratio by more than 2-fold. There was also a functional score improvement (Westvik method) in comparison to the saline-injected diabetic ischemic mice (*p<*0.05). Histologic evaluation demonstrated a nearly 4-fold increase in the number of microvessels and a reduction in apoptotic cells in the WJMSCs *versus* saline injection groups (*p<*0.05) [132]. In a more recent study, Musiał-Wysocka and colleagues [24] compared the effect of human WJMSCs (1 × 10^6^) administered through subcutaneous injection with placebo (PBS) injection and a sham procedure in a mouse model of hind-limb ischemia. Animals treated with WJMSCs showed a reduction in fibrosis and a higher number of proliferating cells. At 21 days, blood flow significantly increased in the WJMSCs-treated group compared with controls (sham and PBS groups). Furthermore, the functional condition of the ischemic hind limbs was improved in the WJMSCs-treated group compared to the control groups [70].

A recent pilot study in humans demonstrated safety, feasibility, and a suggestion of efficacy of combined intra-arterial and intramuscular delivery of WJMSCs in patients with no-option critical limb ischemia [243].

## CONCLUSION

Ischaemic heart disease remains the leading cause of death worldwide [1, 2, 42-48]. CIHF, involving a set of significant events over the lifetime further aggravated by patient´s comorbidities, represents a major unresolved health problem [231]. The mortality rate of CIHF remains high, with about 50% of patients dying within 5 years after the initial diagnosis, which exceeds most types of cancer [203]. The multifaceted and complex nature of human ischemic heart disease is difficult to recapitulate in animal models [232] but studies in animal models of human disease by enabling the ‘bench-to-bedside’ transition of novel therapies are indispensable in advancing human studies. Small-animal models are pivotal in developing novel therapies but may be unable to fully recapitulate the human disease [231]. Large-animal models (in particular swine) have generally strong translational relevance to humans and pave the way for the evaluation of new therapies in humans [79, 223, 225].

WJMSCs, fetal stem cells residing in the umbilical cord matrix (Fig. **1**) represent a unique type of mesenchymal stem cells. In the absence of tumorigenicity, which is a significant concern with embryonic stem cells or induced pluripotent stem cells, WJMSCs exhibit key stem cell features resulting from their fetal origin [101]. WJMSCs are not only multipotent, but they also express several pluripotency markers and cardiomyocyte-specific markers [69, 112]. WJMSCs' low immunogenicity, taken together with their strong anti-inflammatory, and immunomodulatory properties, are important advantages in the context of allogenic cell transfers (Figs. **2** and **3**). WJMSCs can be standardized as an advanced therapy medicinal product and produced in large quantities without ethical concerns. Today, there is consistent evidence from cell/tissue studies (Figs. **3A**, **B**) and from studies in animal models of AMI and CIHF (Table **4**) that WJMSCs can promote myocardial repair and regeneration through several biologically relevant mechanisms that include paracrine actions and cell-to-cell communication. WJMSCs possess the biological potential to address the significant unmet needs of first-generation cell-based therapeutic approaches. The key advantages of WJMSCs include potency, genetic stability, safety, availability in large quantities, and feasibility of standardizing the cell product [54]. Importantly, WJMSCs can be tracked *in vivo* in animals and humans [244] using methods previously established for hematopoietic and mesenchymal bone marrow cells [245]. There is emerging evidence that myocardial uptake of transcoronary-delivered WJMSCs may be several-fold greater than that seen with bone marrow-derived hematopoietic or mesenchymal stem cells [244]. WJMSCs can be combined with other innovative approaches, such as the use of engineered/enhanced cells, cocktails of different cell types, and use of cell-derived exosomes/secretome or preparations of cells and scaffolds (injectable hydrogels, natural products, cardiac-like patches, piezoelectric biomaterials). There is also potential to use WJMSCs as a vector to deliver gene therapies to the zones of myocardial ischemic injury [158, 244, 246-255], as well as to counteract aging-related cardiovascular deterioration [256].

Furthermore, cell-based therapeutic strategies employing WJMSCs are suitable to be combined with structural mechanical interventions such as innovative transcatheter left ventriculoplasty [252].

Recent data from both small- and large-animal models of AMI and CIHF have consistently demonstrated a reduction in infarct size, an increase in cardiac contractility, and improved survival (Table **4**) [104, 125, 136-139, 151, 241], paving the way for WJMSCs ATMP clinical trials [116, 257]. The CIRCULATE-AMI pilot study indicated that WJMSCs transcoronary infusion in large AMI in humans is feasible, safe, and may be associated with a sustained LVEF improvement [258]. Randomized, placebo-controlled, double-blind clinical trials of standardized WJMSCs in AMI (NCT03404063) and CIHF (NCT03418233), employing innovative transcoronary cell transfer [259, 260] and precise evaluation of global and regional myocardial contractility and remodeling [244] will provide appropriately powered human data.

## Figures and Tables

**Table 1:** Key endpoints in major clinical trials in acute myocardial infarction and heart failure with reduced left-ventricular ejection fraction.

**Acute Myocardial Infraction**		
**Therapy**	**Trial**	**Endpoint(s)**
Primary PCI	Stone 2016 [3] Keeley E. [4]; Dalby Ml. [5]	↓ Infarct Size Infarct Size = Mortality Predictor ↓ Mortality ↓ Re-infarction
Aspirin	ISIS-2 [6]; Fuster V. [7]	↓ Mortality ↓ Re-infarction
P2Y_12_ receptor inhibitor	PCI-CURE [8]; Verdoia M. [9]	↓ Mortality ↓ Re-infarction
High-dose statin therapy	MIRACL [10]; Afilalo J. [11]; Navarese EP. [12]; Pan Y. [13]	↓ Mortality ↓ MACE
Beta-blockers	CAPRICORN [14] Joo SJ. [15]	↓ Mortality ↓ Re-infarction in patients with LVEF ≤40% ↓ MACE in patients with LVEF <50%
ACE inhibitors	SAVE [16], AIRE [17], Køber L. [18], Sleight P. [19]	↓ Mortality ↓ Severe Heart Failure ↓ Heart Failure Progression
ICD in patients with LVEF ≤35%	Moss AJ. [20]	↓ Sudden Cardiac Death
**Heart Failure with Reduced Left-Ventricular Ejection Fraction**		
ACE-I	CONSENSUS [21], SOLVD [22]	↓Mortality ↓Heart Failure Hospitalization
Beta-blocker	MERIT-HF [23], U.S. Carvedilol Heart Failure Study [24], COPERNICUS [25], SENIORS [26], CIBIS-II [27], Cleland J. [28]	↑ LVEF ↓Mortality ↓Heart Failure Hospitalization
MRA	Randomized Aldactone Evaluation Study, EMPHASIS-HF [29, 30]	↓Mortality ↓Heart Failure Hospitalization
SGLT2 inhibitor (dapagliflozin or empagliflozin)	DAPA-HF [31], EMPEROR-Reduced [32]	↓Mortality ↓Heart Failure Hospitalization
Sacubitril/Valsartan	PARADIGM-HF [33] Zhou X. [34]	↓CV Mortality ↓Heart Failure Progression ↑ LVEF ↓ LV Adverse Remodeling (LVEDD, LVEDVI) ↓ MACE ↓ Heart Failure Hospitalization
Diuretics in patients with signs and/or symptoms of congestion	Faris R. [35]	↓ Mortality ↓ Heart Failure Hospitalization ↑ Exercise Capacity
ARB	CHARM-Alternative [36]	↓ Cardiovascular mortality ↓ Heart Failure Hospitalization
ICD	SCD-HeFT [37]	↓ Mortality
CRT D/P - patients with sinus rhythm, LBBB, QRS width >150 ms	CARE-HF [38] REVERSE [39] COMPANION [40] MADIT-CRT [41]	↑ LVEF ↓ LV Adverse Remodeling (LVESVI) ↓ Mortality ↓ LV Adverse Remodeling (LVESVI) ↓ Heart Failure Worsening ↓ Mortality ↓ Heart Failure Hospitalization ↑ LVEF ↓ LV Adverse Remodeling (LVEDD) ↓ Mortality

**Abbreviations:** ACE-I = angiotensin-converting enzyme inhibitor; LVEF = left ventricle ejection fraction; MRA = mineralocorticoid receptor antagonist; SGLT2 = Sodium-glucose co-transporter 2; MACE = major adverse cardiovascular event; CV = cardiovascular; LVEDD = left ventricle end-diastolic diameter; LVEDVI = indexed left ventricle end-diastolic volume; ARB = angiotensin-receptor blocker; ICD = implantable cardioverter-defibrillator; CRT D/P = cardiac resynchronization therapy - defibrillator/pacemaker; LBBB = left bundle branch block; QRS = Q, R, and S waves of an ECG; LVESVI = left ventricle end-systolic volume index; PCI = percutaneous coronary intervention; AMI = acute myocardial infarction; NB. ↓Mortality = ↑survival cf. (Table **4**).

**Table 2:** Comparison of fundamental characteristics of WJMSCs *vs.* fibroblasts.

**Characteristics**	**WJMSCs**	**Fibroblasts**
Morphology	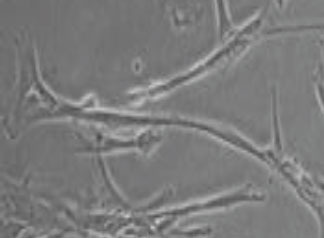 Thin, elongated, spindle-shaped [123]	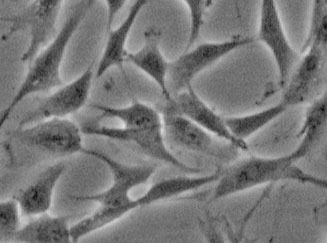 Stellate-shaped [123]
Source	Naturally present in human umbilical cord - arranged in a concentric fashion with their long axis at right angle to the long axis of the cord [66, 67, 69, 124, 125]	Naturally present in human connective tissue [126]
Stemness markers OCT-4 SOX-2 c-MYC NANOG	PRESENT [67-69, 81]	ABSENT [127]
Progenitor cell marker STRO-1	PRESENT [68]	ABSENT [128]
Surface markers (- absent / + present)	- CD 271 + CD 31 + CD 146 + VE-Cadherin - FSP-1 [77]	+ CD 271 - CD 31 - CD 146 - VE-Cadherin + FSP-1 [77]
Differentiation potential	HIGH [77]	RESTRICTED [77]

**Abbreviations:** OCT-4 - octamer-binding transcription factor 4; SOX-2 - SRY (sex determining region Y)-box 2; c-MYC - MYC proto-oncogene, bHLH transcription factor; NANOG - homeobox protein NANOG; STRO-1 - stem cell antigen STRO-1; FSP-1 - fibroblast-specific protein 1.

**Table 3:** Effects of WJMSCs on cell-to-cell mechanisms of infarct healing and remodel.

**-**	**Key Cellular Players**	**WJMSCs Action**	**WJMSCs Effect**	**References**
**AMI**	M1/M2 macrophages	Switch to M2 enhancement	Reduced overt inflammation	[131, 141, 142]
	N1/N2 neutrophiles	Switch towards N2 dominance	Reduced overt inflammation	[143]
	NK cells	Suppression	Inhibition of cytotoxicity	[92, 142, 144, 145]
	Fibroblast-cardiomyocyte crosstalk	Fibroblast replacement	Cardiomyocyte enhancement	[146]
	T-helper cells	Inhibition of differentiation	Reduced inflammation	[142, 144]
	T-regulatory cells	Immunomodulation	Reduced inflammation	[142, 147-149]
**CIHF**	Fibroblast: interstitial fibrosis	↓ Fibrosis ↓ Apoptosis ↓ Hypertrophy ↑ Connexin 43 expression	↑ Cardiomyocyte preservation	[85, 141, 150, 151]
	Cardiomyocyte: apoptosis, hypertrophy, connectivity			

**Note:** See text and Fig. (**3**) for cytokine and transductory molecular mechanisms.

**Table 4:** WJMSCs stimulation of myocardial repair and regeneration: evidence in animal models.

-	**Animal**	**Experimental Protocol**	**WJMSCs Dose**	**Delivery Timing**	**Delivery** **Method**	**Cardiac** **Function Evaluation**	**WJMSCs Effect**	**Comment**
**AMI**								
AMI Small- animal models	Mice [138]	6 groups (*n=*6 each): Placebo* im. WJMSCs im. BM-MSCs im. Placebo* iv. WJMSCs iv. BM-MSCs iv.	0.5 x 10^6^ cells (human WJMSCs)	15 minutes after LAD ligation	Peri-infarct (i.m.) *vs* Intravenous (i.v.)	ECHO - B/L (ie., before MI induction) - 2 weeks after cell transplantation	Peri-infarct WJMSCs injections effective *vs.* Placebo ↑ FS by 40% WJMSCs *vs.* Placebo* (*p<*0.01) WJMSCs>BMSCs ↑ number capillaries in WJMSCs-treated	i.v. therapy ineffective (both WJMSCs and BM-MSCs) WJMSCs expression of α-cardiac actin, cardiac troponin T, α-myosin heavy chain WJMSCs detectable for ≥ 2 weeks
	Mice [137]	WJMSCs (*n=*5) *vs* Placebo** (*n=*10) *vs* Nonmanipulated control group (*n=*10)	0.2 x 10^6^ cells (human WJMSCs)	Following LAD ligation	Peri-infarct (i.m.) injections	ECHO 7 days after AMI 14 days after AMI	14 days after AMI peri-infarct WJMSCs injections effective *vs.* Placebo ↑ EF ↑ FS ↓ LVEDD ↓ LVESD ↑ WT ↓ LV dilation (%) (*p<*0.05 for all) New capillary-like structures in WJMSCs-treated	-
	Rat [125]	WJMSCs (*n=*15) i.v. 3 days after MI *vs* Group after MI (*n=*15) without treatment *vs* WJMSCs (*n=*15) i.v. (no MI induction) *vs* Control nonmanipulated (*n=*15)	5 x 10^6^ cells (human WJMSCs)	3 days after AMI induction	i.v.	ECHO	WJMSCs injections *vs*. AMI without treatment: ↑ LVEF by absolute 9% ↓ LVEDV ↓ LVESV (*p<*0.05 for all) ↑ Number capillaries in WJMSCs-treated	↑ survival with WJMSCs WJMSCs differentiation into cardiomyocyte-like cells: cTnT, α-smooth muscle actin, myosin heavy chain (+)ve WJMSCs detectablefor ≥ 6 weeks
	Rabbit [241]	Intact group (*n =* 7) *vs* AMI controls (*n =* 7) (AMI) *vs* AMI + PBS (*n =* 7) *vs* AMI + WJMSCs (*n =* 7) *vs* AMI + 5-AZT - conditioned WJMSCs group (*n =* 7)	5 x 10^6^ cells (human WJMSCs)	After 1 h of LAD ligation (permanent LAD ligation)	Subepicardial (i.m.)	ECHO 5 and 30 days after AMI	5 days after AMI: no significant differences between study groups (PBS/WJMSCs/ 5-AZT-conditioned WJMSCs) 30 days after AMI: WJMSCs/5-AZT-conditioned WJMSCs groups *vs* PBS (Placebo) and MI groups ↑ EF by absolute 8% ↑ FS by absolute 11% ↓ LVEDD by 20% ↓ LVESD by 20% (*p<*0.05 for all) ↓ Scar tissue with WJMSCs/5-AZT-conditioned WJMSCs	similar effect of non-conditioned *vs*. 5-AZT conditioned WJMSCs Troponin-I (+)ve and F-actin (+)ve proliferating cells New gap junctions expressed in WJMSCs and 5-AZT-conditioned WJMSCs groups
AMI/ Post-AMI Large- animal models	Mini**-**swine [151]	3 groups: WJMSCs high dose (*n=*4) *vs.* WJMSCs low dose (*n=*4) *vs.* Placebo*	High dose: 2x 1.5 x 10^6^ cells/kg Low dose: 2x 0.5 x10^6^ cells/kg (porcine WJMSCs)	120 min after LAD ligation and 4 weeks after MI	i.v.	ECHO: before MI induction *vs.* acute MI after surgery *vs.* at 1 *vs.* 4 *vs.* 8 weeks after MI SPECT/PET 1 *vs.* 4 *vs.* 8 weeks after MI	8 weeks after MI: ↑ FS WJMSCs high dose *vs.* PBS group (*p<*0.05) ↑LVEF - WJMSCs high and low dose *vs.* PBS ↑ LV wall motion - WJMSCs high and low dose *vs.* PBS ↓ LV nonviable myocardium area after MI - WJMSCs high and low dose *vs.* PBS (*p<*0.01) ↓ Infarct area - WJMSCs high and low dose *vs.* PBS (*p<*0.01) ↓ LV fibrosis area - WJMSCs high and low dose *vs.* PBS (*p<*0.01)	↓ inflammation: ↓ TNF-α in infarct area: WJMSCs low dose *vs.* PBS (*p<*0.05) ↓ IL-6 in the border area: WJMSCs low dose *vs.* PBS (*p<*0.05) ↑angiogenesis: ↑VEGF in the border area: WJMSCs high dose *vs.* PBS (*p<*0.05) ↑PECAM-1 in the infarct and border area: WJMSCs low dose *vs.* PBS (*p<*0.05) ↑ Cx43 expression: WJMSCs high dose *vs.* low-dose of WJMSCs and PBS (*p<*0.05)
AMI/ Post-AMI Large animal models	Mini**-**swine [104]	3 groups (*n=*6 each) WJMSCs *vs.* Placebo* vs. Control (no treatment)	40 x 10^6^ cells (human WJMSCs)	Right after LAD ligation	Peri-infarct (i.m.) (10 injections)	ECHO Before AMI induction *vs.* acute AMI *vs.* at 6 weeks post-AMI	6 weeks after MI: EF (%) ↑ WJMSCs *vs* placebo/control (*p<*0.001) ↓ Infarct area ↑ ∆ WT (%) with WJMSCs *vs* placebo/control (*p<*0.001) ↑ Myocardial perfusion ↓ Apoptosis ↓ Fibrosis with WJMSCs	resident cardiac stem cells recruitment with WJMSCs at 6 weeks new cTnT, vWB, c-kit positive cells, presence
**CIHF**								
Post-AMI Small- animal model	Rat [136]	2 groups WJMSCs (*n=*12) *vs.* Placebo* (*n=*11)	5 x 10^6^ cells (human WJMSCs)	2 weeks after LAD ligation	Peri-infarct injections	ECHO Before transplantation (2 weeks after MI) *vs.* 2 weeks after cell transplantation 4 weeks after cell transplantation	After 2 weeks: ↑ LVEF by absolute 10% with WJMSCs ↓ LVEDD with WJMSCs *vs* P ↓ LVESD with WJMSCs *vs* P (*p<*0.05 for all) ↑ WT ↑ Capillary density ↑ Number of arterioles with WJMSCs *vs* P (*p<*0.05 for all)	WJMSCs present for at least 4 weeks (cTnT, vWF, smooth muscle actin expression) WJMSCs benefit seen at 2 weeks and sustained at 4 weeks
Post-AMI Large- animal model	Swine [139]	2 groups WJMSCs *vs.* Placebo***	30x10^6^ cells i.c. 2x 30x10^6^ cells iv (human WJMSCs)	4 weeks after MI (ameroid constrictor on LCx) 5^th^ and 6^th^ week after surgery	ic. infusion iv. infusion	ECHO Baseline - the day before cell transplantation (4 weeks after MI) *vs.* 4 weeks after WJMScs transplantation (8 weeks after MI)	↑ EF by absolute 11% WJMSCs *vs* placebo (*p* < 0.05) ↑ Thickening fraction in the infarcted LV wall by absolute 5% WJMSCs *vs* placebo (*p* < 0.01) Inhibitoin of LV adverse remodelling: LVEDV and LVESV unchanged with WJMSCs *vs.* ↑ LVEDV and ↑ LVESV (*p* < 0.05) with Placebo ↑ Capillary density in WJMSCs-treated *vs* placebo (*p<*0.01)	↓ Apoptosis (*p<*0.001) ↓ Fibrosis (*p<*0.01) with WJMSCs

**Note:** Placebo*- PBS -phosphate-buffered saline, Placebo** - bovine serum albumine in PBS, Placebo***- saline. **Abbreviations:** AMI - acute myocardial infarction, MI - myocardial infarction, WJMSCs - Wharton’s jelly mesenchymal stem cells, 5-AZT - 5-Azacytidine; BM-MSCs - bone marrow mesenchymal stem cells, B/L - baseline, ECHO - transthoracic echocardiography, EF - ejection fraction, FS - fractional shortening, LVEF - left ventricle ejection fraction, LVEDD - left ventricle end-diastolic diameter, LVESD - left ventricle end-systolic diameter, LVEDV - left ventricle end-diastolic volume, LVESV - left ventricle end-systolic volume, LVW - left ventricle wall, WT - wall thickening, vWF - von Willebrand factor, cTnT - cardiac troponin T, PBS- phosphate buffered saline, BSA - bovine serum albumin, DIM - Diabetic ischemic mice, ROS- reactive oxygen species, LCx - left circumflex coronary artery, i.v. intravenous, i.m. intramuscular, i.c. intracardiac, VEGF- vascular endothelial growth factor, PECAM-1 - platelet/endothelial cell adhesion molecule 1 (CD-31), Cx43 - Connexin 43 - the major gap junction protein expressed in the heart, P=placebo.

## Footnotes

1 Under the European Medicines Agency the acronym used is "ATMP"; in the USA the equivalent Food and Drug Administration acronym is "RMAT" (Regenerative Medicine Advanced Therapy Designation).

## LIST OF ABBREVIATIONS

AMI: Acute Myocardial Infarction
ANGPT-1: Angiopoietin-1
ATMP: Advanced Therapy Medicinal Product
CIHF: Chronic Ischemic Heart Failure
LV: Left Ventricle
LVEDD: Left Ventricular End-diastolic Diameter
LVEDV: Left Ventricular End-diastolic Volume
LVEF: Left Ventricular Ejection Fraction
LVESD: Left Ventricular End-systolic Diameter
LVESV: Left Ventricular End-systolic Volume
LVFS: Left Ventricular Fractional Shortening
MHC: Major Histocompatibility Complex
VEGF: Vascular Endothelial Growth Factor
WJMSCs: Wharton’s Jelly Mesenchymal Stem Cells
